# Cryptotanshinone Alleviates Oxidative Stress and Reduces the Level of Abnormally Aggregated Protein in *Caenorhabditis elegans* AD Models

**DOI:** 10.3390/ijms231710030

**Published:** 2022-09-02

**Authors:** Wen-Bo Cui, Zong-Ping Zhang, Xue Bai, Shan-Shan Wang, Xiao-Han Chen, Xu Liu, Pan-Jie Su, De-Juan Zhi, Dong-Qing Fei, Zhan-Xin Zhang, Dong-Sheng Wang

**Affiliations:** School of Pharmacy, State Key Laboratory of Applied Organic Chemistry, Lanzhou University, Lanzhou 730000, China

**Keywords:** Alzheimer’s disease, cryptotanshinone, *Caenorhabditis elegans*, Aβ, oxidative stress

## Abstract

Alzheimer’s disease (AD) is one of the leading causes of dementia. As the first common neurodegenerative disease, there are no effective drugs that can reverse the progression. The present study is to report the anti-AD effect of cryptotanshinone (CTS), a natural product isolated from *Salvia castanea*. It is found that it can alleviate AD-like features associated with Aβ_1-42_ toxicity in muscle cells as well as neuronal cells of *Caenorhabditis elegans* (*C. elegans*). Further studies showed that CTS reduced the level of reactive oxygen species (ROS) in nematodes, up-regulated the expression of *sod-3*, and enhanced superoxide dismutase activity. Cryptotanshinone reduced the level of Aβ monomers and highly toxic oligomers in *C. elegans* while inhibiting the abnormal aggregation of polyglutamine protein. In addition, CTS upregulated the expression of *hsp-16.2* and downregulated the expression of *ace-2*. These results suggested that CTS could alleviate oxidative stress and reduce the level of abnormally aggregated proteins and has the potential to be developed as an anti-AD drug candidate.

## 1. Introduction

Alzheimer’s disease (AD) is a progressive neurodegenerative disease with an insidious onset and is one of the leading causes of dementia [1]. The main pathological hallmarks of AD are the formation of senile plaques (SP) as well as neurofibrillary tangles (NFT) in the brain, with clinical manifestations of cognitive decline, persistent memory loss, and language impairment [2], personality changes [3], and other psychiatric-related symptoms.

The incidence of AD is closely linked with the increase in age. The global prevalence of dementia among people over 60 is estimated to be 3.9%. The prevalence is almost doubled every 5 years among people after 65 years old. In developed countries, almost one in ten elderly people will be affected by dementia. The overall prevalence of AD in developing countries is 3.4% [2]. The occurrence of AD can also significantly shorten life expectancy, raise the disability rate, and cause a decline in the quality of life. It has been reported that AD and vascular dementia, such as the malignancy tumor, are major risk factors for death in people over the age of 75 [4]. With the number of AD patients increasing year by year, the burden and harm caused to families and society are getting worse [5,6].

Alzheimer’s disease can be classified as familial or sporadic according to the mode of onset. Familial AD usually has an early onset and is associated with mutations in the amyloid precursor protein (APP), presenilin 1, and presenilin 2 genes [7]. Otherwise, apolipoprotein E on chromosome 19 is the strongest genetic risk factor for AD [8]. The study found that exposure to depression, high blood pressure, and diabetes mellitus type 2 increased the risk of developing AD. Smoking, alcohol consumption, obesity, and stress are all important factors in the risk of AD [9]. 

The pathogenesis of AD is extremely complex. There is no accurate conclusion so far, it is mainly thought to be the result of the interaction of genetic and environmental factors. The current popular hypotheses mainly include the Aβ cascade hypothesis, cholinergic hypothesis, tau protein hypothesis, inflammation hypothesis, oxidative stress damage, etc. [10,11,12]. The Aβ cascade hypothesis suggests that the amyloid β-protein (Aβ), a major component of SP, is a key factor in the pathogenesis of AD. The Aβ monomers are produced by APP via cleaving enzymatically by the β- and γ-secretase enzymes. Aβ aggregates further form oligomers, fibrils, and plaques [13]. Among them, Aβ oligomers are considered to be the main toxic components [7], and the fibrils can also act as a reservoir of toxic oligomers through fracture and secondary nucleation [14]. 

Traditional anti-AD drugs such as NMDA receptor (N-methyl-D-aspartic acid receptor) antagonists (memantine, etc.) and cholinesterase inhibitors (donepezil, galantamine, etc.) are symptomatic treatments. They can only be used to improve cognitive and memory function, cannot fundamentally reverse the onset of AD [15], and are often expensive and have side effects. In addition, most of the drugs for AD that have entered clinical trials for decades have failed [16]. Even the very few drugs that make it to market are controversial in terms of efficacy and side effects. Lessons from previous failures in anti-AD drug development suggest that effective AD therapy should include multiple targets to reduce the incidence of multiple risk factors for disease onset and progression [17]. Therefore, the “multitarget-directed ligand” design strategy (i.e., one drug modulates multiple targets simultaneously) offers a new approach to treating AD [18,19]. 

**Figure 1:**
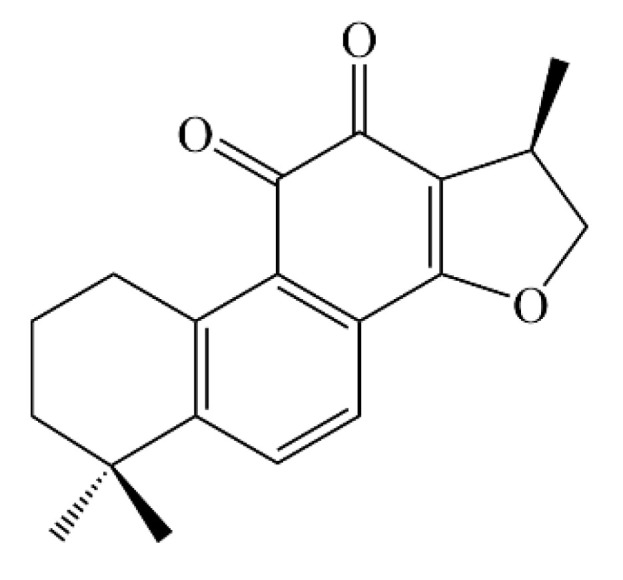
Chemical structure of cryptotanshinone.

As a natural treasure trove for new drug discovery, small molecules in Chinese herbs offer more possibilities for anti-AD drug discovery. Compared with chemically synthesized drugs, natural drugs tend to have a greater range of safety, fewer adverse effects, and multiple target activities. Cryptotanshinone (CTS) exists in many kinds of herbs in *Salvia* (Figure 1), such as *Salvia miltiorrhiza* and *Salvia castanea*. It has manifested a wide range of pharmacological activities, including anti-cancer, anti-inflammation, anti-metabolic disorder, cardioprotective, and neuroprotective activity [20]. *Caenorhabditis elegans*, as a common model organism, has been widely used in drug screening and the mechanism research of aging-related diseases, especially neurodegenerative diseases [21]. It has the advantages of being inexpensive, easy to cultivate, having a short growth cycle, obtaining a large number of individuals with the same genetic background, and being highly conserved in signaling pathways with higher organisms [22]. It provides an ideal model for the study of AD.

The present study was conducted to investigate the effects of CTS on Aβ protein toxicity and protein self-levels using the *C. elegans* model and found that CTS regulates nematode oxidative stress and abnormal protein folding through multiple mechanisms.

## 2. Results

### 2.1. Cryptotanshinone Delayed Progressive Paralysis of AD C. elegans and Decreased the Ratio of Exogenous Serotonin-Induced Acute Paralysis 

**Figure 2:**
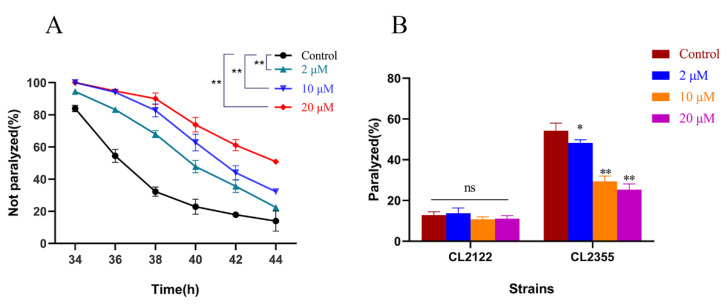
CTS retards Aβ-induced AD-like pathological features in transgenic nematodes. (**A**) CTS delayed Aβ-induced CL4176 paralysis in a concentration-dependent manner. Sixty nematodes were in each treatment group. (**B**) CTS reduced the sensitivity of CL2355 to exogenous serotonin while not affecting its transgenic control CL2122. Thirty nematodes were in each treatment group. Briefly, 0.1% DMSO was used as the control. The data represent the Mean ± SD. “ns” means no statistical significance (* *p* < 0.05, ** *p* < 0.01 vs. control).

Aβ-induced neurotoxicity is one of the most important features of Alzheimer’s disease [23]. To further evaluate the effect of CTS treatment on Aβ-induced pathology, a temperature-sensitive *C. elegans* strain CL4716 that expresses Aβ in muscle cells was employed. As shown in Figure 2A, CTS significantly delayed the Aβ-induced paralysis of CL4176 nematodes. After 44 h of the upregulation of the temperature, 51% of nematodes were still unparalyzed in the 20 μM CTS-treated group, while only 14% of unparalyzed nematodes remained in the 0.1% DMSO-treated group. The result indicated that CTS protected against Aβ toxicity in muscle cells. 

Transgenic strain CL2355 expresses Aβ protein in Pan-neurons, which is more compliant with the pathological features of AD [24]. In the serotonin sensitivity assay, the ratio of paralysis in the CL2355 strain was decreased in a dose-dependent manner by CTS. The percentage of paralysis was reduced by 53% after treatment with 20 μM CTS. On the contrary, serotonin and CTS treatment did not affect the control CL2122 strain the same as the CL2355 Aβ transgenic strain (Figure 2B). Therefore, CTS could alleviate the 5-HT hypersensitivity of nematodes caused by the over-expression of Aβ in the nerve cells.

### 2.2. Cryptotanshinone Decreased the Level of Aβ Monomers and Oligomers in the Transgenic C. elegans

**Figure 3:**
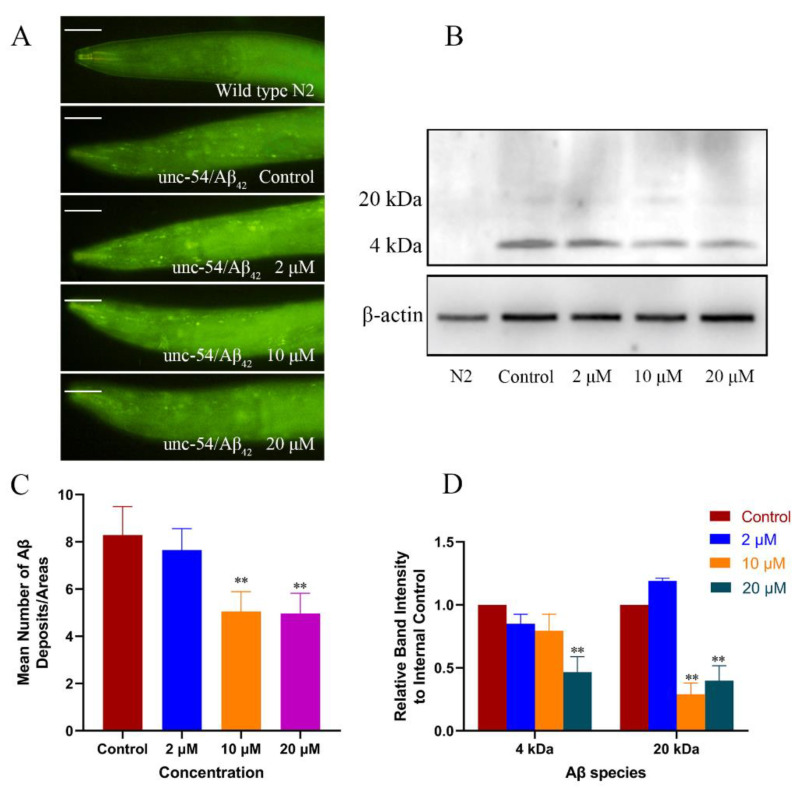
CTS decreased Aβ deposits and reduced Aβ oligomers in AD worms. (**A**) CTS significantly reduced the ThS-stained Aβ deposition spots in CL2006 *C. elegans*. The scale bar is 50 μm. (**B**) Western blotting analysis of Aβ species and relative content in CL4176 nematodes. (**C**) Quantitative analysis of the number of deposited spots per unit area in the head region of nematodes after ThS staining. At least 30 nematodes were counted in each group. (**D**) Quantitative measurement of the relative density of Aβ to β-actin expression. Wild-type N2 strain was used as transgenic control. Briefly, 0.1% DMSO was used as the negative control (** *p* < 0.01 vs. control).

CTS alleviated the Aβ-induced toxic response in the transgenic nematodes, but whether it can reduce Aβ levels is not yet known. Hence, ThS staining assay and Western blotting were used to detect the effect of CTS on the levels of Aβ monomers and oligomers in nematode cells. The transgenic strain CL2006 continuously expressed Aβ_1–42_ in muscle cells was used to determine the effect of CTS on Aβ deposits by counting the number of deposits on the head region after staining with ThS. The results, as shown in Figure 3A,C, indicated that CTS reduced the Aβ_1–42_ deposits. On the other hand, the Western blotting detection of the effect of CTS on the 4 kD monomer and 20 kD oligomers of Aβ was completed. The results showed (Figure 3B,D) that the levels of Aβ monomers and oligomers in CL4176 nematodes treated with 20 μM CTS were reduced by 53% and 60%, respectively, compared with the control. 

**Figure 4:**
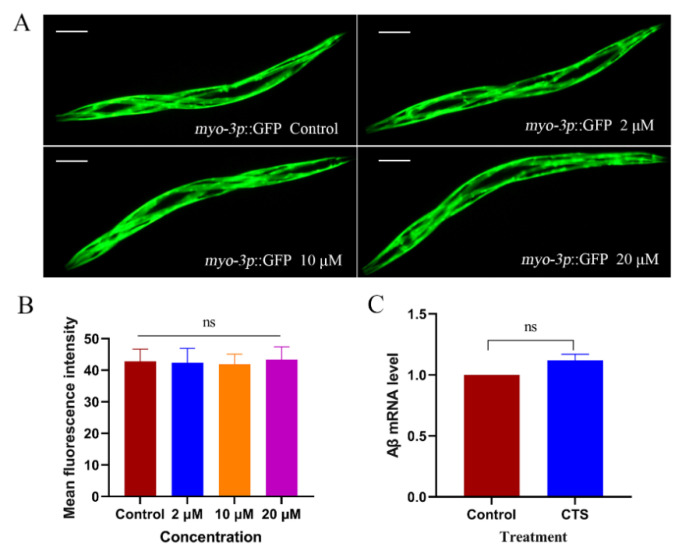
Effect of CTS on exogenous GFP protein expression and Aβ transcript levels. (**A**) Representative fluorescence images of CL2179 *C. elegans* with myo-3p::GFP. The scale bar is 100 μm. (**B**) Graphical representation for the fluorescence intensity of myo-3p::GFP of a transgenic *C. elegans* as quantified using ImageJ. (**C**) Effects of CTS on transcription level of Aβ in nematode CL4176. CL4176 was incubated at 15 °C to L3, and then the temperature was raised to 25 °C. In total, 0.1% DMSO was used as the control. The concentration of CTS is 20 μM. The transcription level of Aβ mRNA was determined by β-actin. The data represent the Mean ± SD. “ns” means no statistical significance.

CL4176 was transferred into the exogenous Aβ gene resulting in AD-like pathological features in nematodes. Differently, CL2179 was only transferred into GFP instead of Aβ under the same transgenic conditions as CL4176. It was possible to determine whether CTS specifically reduced the level of exogenous Aβ_1-42_ or had an effect on all exogenous proteins by treating this strain with CTS. The result showed that there was no difference between CTS treatment and vehicle control (Figure 4A,B). This suggested that CTS reduced specifically the level of Aβ monomers and oligomers. To further verify whether this effect of CTS was related to its influence on the transcript level of Aβ, this study examined the Aβ transcript level of CL4176 after 30 h of induction at 25 °C. As shown in Figure 4C, the Aβ mRNA transcript levels in CTS-treated AD nematodes did not differ from the control, indicating that CTS did not reduce Aβ protein levels by decreasing Aβ transcript levels.

### 2.3. Cryptotanshinone Upregulated hsp-16.2 Expression and Reduces Abnormal Polyglutamine Protein Aggregation Levels in Nematodes

**Figure 5:**
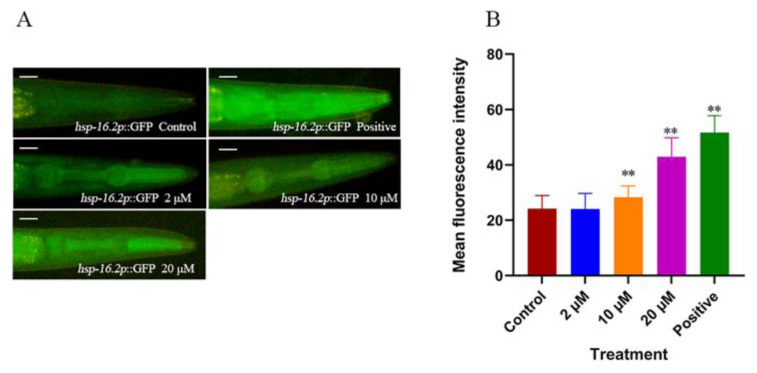
Effect of CTS on the expression of *hsp-16.2* of nematodes (**A**) Fluorescent images of TJ375 nematodes. The scale bar is 50 μm. (**B**) Quantitative analysis of fluorescence intensity of each treatment group. The control is 0.1% DMSO. Heat stress at 35 °C for 30 min was used as the positive control. At least 30 nematodes were counted in each group (** *p* < 0.01 vs. control).

In response to environmental stress and age-related protein folding disorders, the nuclear transcription factor HSF-1 can suppress the toxicity of aberrant proteins by upregulating target chaperone genes [25]. *hsp-16.2* is located downstream of HSF-1 and encodes Heat Shock Proteins (HSPs) [26]. In this study, the effect of CTS on nematode *hsp-16.2* expression was observed using fluorescence microscopy, and the results were shown in Figure 5. A total of 10 μM and 20 μM CTS upregulated *hsp-16.2* expression by 17% and 77%, respectively, and incubation at 35 °C for 30 min as a positive control upregulated *hsp-16.2* expression by 114%.

**Figure 6:**
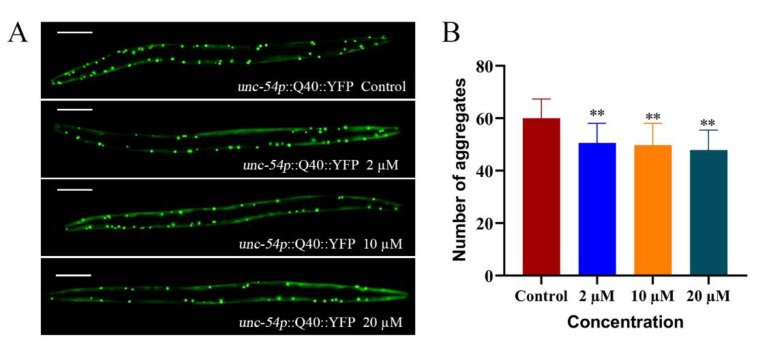
CTS alleviates the abnormal aggregation of polyglutamine protein in AM141 nematodes. The scale bar is 100 μm. (**A**) Fluorescent images of aggregation of Q40 of AM141 nematodes. The scale bar is 100 μm. (**B**) Quantitative analysis data for Q40 fluorescent spots of AM141 nematodes. In total, 0.1% DMSO was used as the control. The nematodes were treated at 20 °C for 72 h. At least 30 fluorescent images per group. Results are expressed as mean ± SD (** *p* < 0.01).

The aggregation of specific proteins is associated with specific neurodegenerative diseases. The transgenic strain AM141 expresses polypeptide with 40 glutamine residues fused to a yellow fluorescent protein (Q40-YFP) [21]. It is commonly used to detect pathological features related to protein deposition and aggregation, and to simulate polyglutamine protein aggregation in Huntington’s chorea (HD) [27]. After 72 h of the induction of the AM141 nematodes, fluorescence aggregation was significantly increased in the control, while fluorescence aggregation became significantly less after treatment with different concentrations of CTS (Figure 6). This suggested CTS can alleviate the abnormal aggregation levels of polyglutamine protein in AM141 nematodes. It is further proved that CTS can specifically inhibit the aggregation of abnormal proteins like Aβ and may have the same therapeutic effect on diseases based on misfolded proteins as the etiology. 

### 2.4. Cryptotanshinone Promoted the Activity of SOD and Alleviated Oxidative Stress in C. elegans

**Figure 7:**
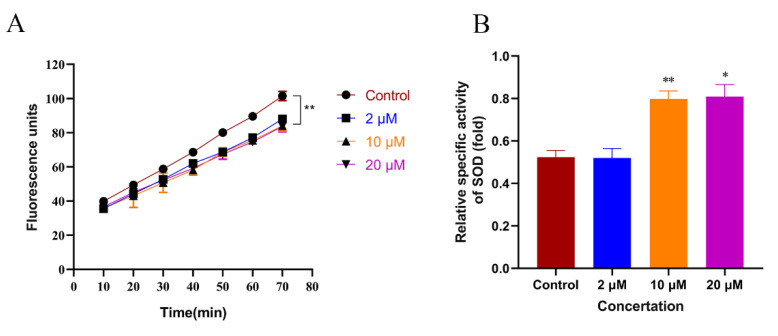
CTS reduces ROS levels and enhances SOD viability in CL4176 nematodes. (**A**) Effect of CTS on ROS fluorescence of *C. elegans* over time. (**B**) CTS enhances the CL4176 nematode activity of SOD. The vertical coordinate is the SOD activity unit of nematode extracts under different treatment conditions. Briefly, 0.1% DMSO was used as the control. All values are expressed as mean ± SD. (* *p* < 0.05, ** *p* < 0.01 vs. control).

The accumulation of Aβ leads to mitochondrial dysfunction and ROS release. Excessive ROS accumulation aggravates Aβ toxicity and promotes neuroinflammation, leading to neuronal apoptosis. Therefore, oxidative stress plays an important role in Aβ-induced toxicity [28]. The present study used the H2DCF-DA kit to measure the ROS levels in AD nematodes. As shown in Figure 7A, compared with the control group, the ROS was reduced by 13%, 17%, and 18% in the 2, 10, and 20 μM CTS-treated groups, respectively (Figure 7A). Antioxidant enzyme systems in organisms, especially superoxide dismutase (SOD), scavenge different ROSs and play an important role in mitigating oxidative stress and aging at the cellular level [29]. This study examined the SOD activity in CL4176 nematodes after CTS treatment. As shown in Figure 7B, compared with the control, the activity of SOD increased by 35% in nematodes treated with 20 μM CTS. Therefore, CTS could upregulate the activity of SOD and reduce ROS accumulation, improving cellular damage caused by oxidative stress in nematodes. 

**Figure 8:**
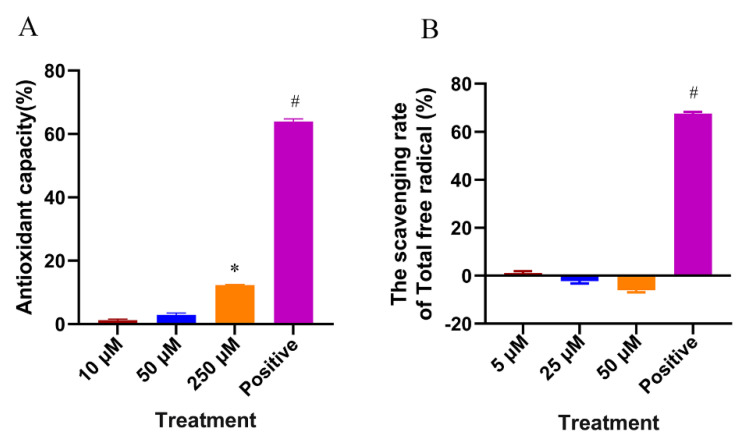
In vitro antioxidant capacity of CTS. (**A**) Scavenging effect of CTS on DPPH free radicals. Positive control was 30 μM L-ascorbic acid. (**B**) Total in vitro antioxidant capacity of CTS. Positive control was 30 μM Trolox. Bars with a hash (#) or asterisk (*) indicate statistical significance (*p* < 0.05, mean ± SD, n = 3).

In this study, the in vitro free radical scavenging and antioxidant capacity of CTS were determined. As shown in Figure 8A, the positive control L-ascorbic acid (L-Aa) had a strong free radical scavenging ability, while CTS did not have a free radical scavenging ability. The high concentration group even showed a “negative” free radical scavenging effect due to the color interference of CTS itself. As shown in Figure 8B, 250 μM CTS exhibited an extremely weak antioxidant capacity of only 12% in vitro. During in vivo dosing, it is often difficult to achieve such high concentrations (250 μM) of CTS. In total, 50 μM of CTS has almost no antioxidant capacity anymore, while the positive control 30 μM of Trolox exhibited 64% antioxidant capacity. This suggested that the effect of CTS in alleviating oxidative stress in nematodes is not dependent on direct antioxidant action. 

### 2.5. Cryptotanshinone Upregulated the Expression of Superoxide Dismutase Gene sod-3 and Downregulated the Expression of Cholinesterase Gene ace-2

**Figure 9:**
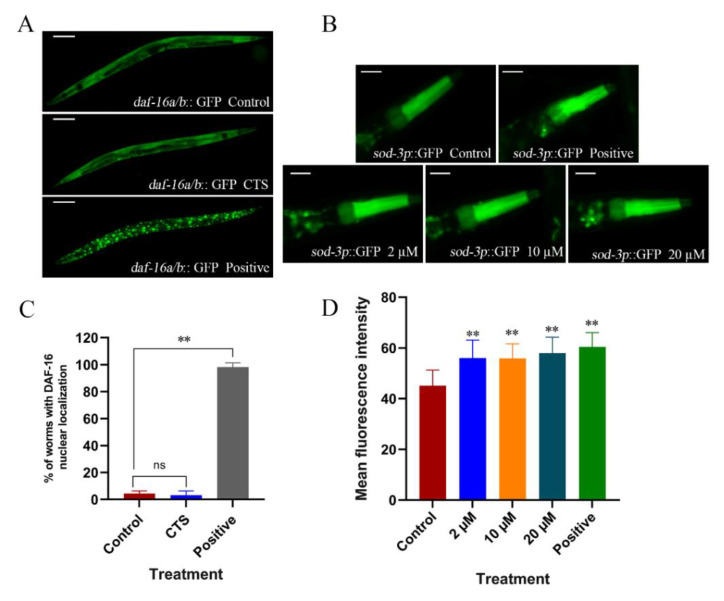
Effect of CTS on the nuclear translocation of DAF-16::GFP and the expression of *sod-3p*::GFP in *C. elegans*. (**A**) Fluorescence images of TJ356 nematode. Briefly, 0.1% DMSO was used as the control. The concentration of CTS was 20 μM. Incubate at 37 °C for 30 min as a positive control. The scale bar is 100 μm. (**B**) Statistics on the effects of different treatments on nematode DAF-16 nuclear translocation. Statistics from three independent replicate trials. (**C**) Fluorescence images of CF1553 nematode. The negative control was 0.1% DMSO. In total, 20 μM juglone treatment for 24 h was used as a positive control. The scale bar is 50 μm. (**D**) Quantitative analysis of fluorescence intensity of CF1553 nematode. Each group counted at least 30 fluorescent images. “ns” means no statistical significance (** *p* < 0.01 vs. control).

In *C. elegans*, DAF-16 is a homologous transcription factor of mammalian FOXO. It is activated under stress conditions and initiates the expression of different protective genes, including antioxidant proteins, such as superoxide dismutase or glutathione S-transferase [30,31]. The *sod-3* of *C. elegans* locates downstream of DAF-16. It belongs to the SOD family and encodes the SOD-3 protein, which plays a crucial role in the nematode’s response to antioxidant stress. In this study, the effect of CTS on DAF-16 translocation and *sod-3* expression was investigated by microfluorimetry. The results showed (Figure 9A,C) that CTS at 20 μM could not promote DAF-16 into nuclear translocation, while the positive control group of heat stress translocated significantly. This suggested that CTS cannot activate the DAF-16 transcription factor to play a role in alleviating oxidative stress. Unexpectedly, CTS treatment at 2, 10, and 20 μM upregulated *sod-3* expression in CF1553 nematodes by 24%, 24%, and 28% compared with the control, respectively. A total of 20 μM juglone upregulated *sod-3* expression by 34% (Figure 9B,D). The upregulation of the *sod-3* expression level in nematodes by CTS may be related to its enhancement of SOD activity and reduction in ROS levels. 

**Figure 10:**
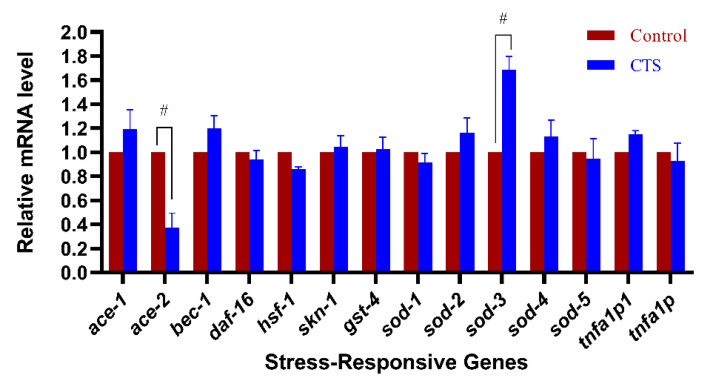
Effect of CTS on the expression level of AD-related genes. The synchronized CL4176 nematodes were cultured for 15 h and then the temperature was raised to 25 °C for another 30 h. The control is 0.1% DMSO. The concentration of CTS is 20 μM. A hash (#) indicates a difference in gene expression compared with the control group (*p* < 0.05).

Considering that AD is a complex neurodegenerative disease and compounds often involve multiple mechanisms in influencing the pathological features of AD, the expression levels of several classical transcription factors and AD-related genes in transgenic nematodes were examined using the qRT-PCR technique (Figure 10). 

The experimental results showed that CTS significantly upregulated the expression of *sod-3*. This is consistent with the results of microscopic fluorescence quantification. In addition, CTS downregulated the expression of the cholinesterase gene *ace-2*, while it did not affect the expression of *ace-1*, *bec-1*, *daf-16*, *hsf-1*, *skn-1*, *sod-1*, *sod-2*, *sod-4*, *sod-5*, *tnfa1p1*, *and tnfa1p*. Anyhow, the roles of CTS on the upregulation of *sod-3* and the downregulation of *ace-2* expression provided beneficial protective effects for AD nematodes. 

## 3. Discussion

Alzheimer’s disease is a complex neurodegenerative disease involving multiple pathogenic factors, and Aβ deposition is one of its main pathological features [32]. Although each of the constituent forms of Aβ has some toxicity, there is a growing consensus that amyloid oligomers are more toxic than monomers or fibrils [33]. Misfolded protein aggregates are therefore considered important targets for the treatment of AD. Starting from Aβ provides us with an effective entry point for anti-AD drug screening and discovery. This study explored the anti-AD effect of CTS and found that it significantly reduced the proportion of Aβ-induced paralysis in CL4176 nematodes and attenuated the hypersensitivity of CL2355 nematodes to exogenous 5-HT. This result indicated that CTS not only reduced the toxicity of Aβ in nematode muscle cells but also alleviated the toxicity of Aβ expressed in neurons, suggesting that it may have the potential to be developed into an anti-AD agent.

Further studies revealed that CTS reduced the number of Aβ deposition spots without affecting the level of Aβ transcription while down-regulating the level of Aβ monomers and oligomers. The nematodes treated with different concentrations of CTS did not affect exogenous GFP expression, suggesting that CTS does not have a general inhibitory effect on exogenous proteins transferred to nematodes. It is worth noting that Mei et al. [34] found that CTS was able to induce APP metabolism in the α-secretase pathway by upregulating the α-secretase activity responsible for shearing the β-amyloid precursor protein (APP) fragment, thereby increasing the release of sAPPα and decreasing Aβ levels. It is suggested that CTS may promote α-secretase activation by activating the phosphatidylinositol 3-kinase (PI3K) pathway on cortical neurons through the activation of α-secretase [35]. Furthermore, CTS can also promote the translocation of ADAM10 and PKC to the cell membrane, resulting in the cleavage of APP at the site where enzymes are secreted, thus releasing sAPPα into the extracellular environment [36]. However, the transgenic AD nematodes used in this experiment were directly transferred to the human Aβ gene, and the effect of CTS on Aβ transcript levels was excluded by qRT-PCR, so this inhibitory effect of CTS on Aβ in AD nematodes must occur at some point after the Aβ production. This research is the first time investigating the role of CTS in scavenging Aβ and using *C. elegans* as a model.

Abnormal protein folding and deposition are common features of neurodegenerative diseases [37]. The present study found the same alleviating effect of CTS on the abnormal aggregation of polyglutamine protein. It suggests that CTS may have a therapeutic effect on similar neurodegenerative diseases caused by protein misfolding or aggregation. In a similar vein, ginsenosides have previously been reported to reduce the oligomeric levels of Aβ proteins by activating the HSF-1 molecular signaling pathway, while inhibiting the expression of the Q40::YFP protein in AM141 nematodes [38]. Wang et al. found that ursolic acid reduced β-amyloid levels by upregulating the proteasome activity of AD nematodes, while it also had an inhibitory effect on human-derived α-synuclein transferred in OW13 nematodes [39]. HSPs play a key role in preventing the formation of toxic aggregates of abnormal proteins and assisting in their degradation [40]. It has been reported that *hsp-16.2* can bind directly to Aβ in vitro and affect its oligomerization to reduce its toxicity [41]. In the research, CTS upregulated the expression of the heat shock protein gene *hsp-16.2*, which may provide additional beneficial effects to alleviate the abnormal folding of Aβ protein in nematodes.

Oxidative stress is thought to play an important role in the pathogenesis of AD. Targeting cellular levels of oxidative stress has become an important tool in the treatment of AD. Cryptotanshinone significantly reduced the Aβ-induced, elevated ROS levels in CL4176 nematodes. The in vitro antioxidant experiments revealed that CTS itself does not contain free radical scavenging capacity and the total antioxidant capacity was extremely weak. The in vivo administration concentration of nematodes no longer has antioxidant capacity in vitro. This suggests that CTS must have reduced ROS levels in nematodes through other indirect regulations. The study further examined the activity of SOD after administration and found that the SOD activity was significantly upregulated, which may play a key role in the antioxidant process of CTS.

It has been shown that the insulin/insulin growth factor signaling (IIS) pathway is a highly conserved developmental and aging controller in living organisms [42]. DAF-16, a FOXO family homologous transcription factor, is a major target of IIS [43]. It regulates lifespan and various stress responses in nematodes, drosophila, and mammals [44]. In this study, the effect of CTS on DAF-16 translocation was evaluated using the TJ356 worms. The results showed that CTS could not activate its nuclear translocation, but unexpectedly, CTS upregulated the expression of the *sod-3* gene, which is known to be located downstream of *daf-16* and regulates the expression of the superoxide dismutase SOD-3 [45]. The upregulation of *sod-3* expression is consistent with the enhanced SOD enzyme activity. Cryptotanshinone did not activate the DAF-16 translocation but upregulated the expression of *sod-3*, which is different from the previous report [46] in that the upregulation of *sod-3* expression is dependent on the activation of DAF-16. It needs to be further verified.

Decreased acetylcholine levels are one of the important pathological features of AD patients. The main role of acetylcholinesterase (AchE) is to break down acetylcholine. AchE is involved as a “molecular chaperone” in the in vitro aggregation of β-amyloid, increasing protofibrillar assembly and Aβ neurotoxicity [47]. It has been reported that Aβ increases AchE expression in neuroblastoma cells [48], which in turn promotes Aβ aggregation, increasing Aβ toxicity [49]. *C. elegans* has several AchE genes (*ace-1*, *ace-2*, *ace-3*, etc.), with the expression products of *ace-1* and *ace-2* accounting for 95% of the overall enzyme activity [50]. Drugs that inhibit AchE activity are widely used in the treatment of AD with good results [50]. Wong et al. [51] found that CTS is a non-competitive inhibitor of AchE using in vitro enzyme activity assays and molecular docking techniques. In the present study, CTS significantly down-regulated the expression of the AchE gene *ace-2*, which may be related to CTS alleviating the level of Aβ aggregation and toxicity in AD nematodes. Overall, the downregulation of AchE gene expression by CTS contributed to the improvement of pathology symptoms in AD nematodes.

## 4. Materials and Methods

### 4.1. Strains and Maintenance

All nematodes employed in this study were obtained from the Caenorhabditis Genetics Center (CGC). *C. elegans* of wild-type Bristol N2; CL4176, dvIs27 [*myo-3p*::Abeta (1-42)::let-851 3′UTR) + rol-6(su1006)]; CL2122, dvIs15 [(pPD30.38) unc-54(vector) + (pCL26) mtl-2::GFP]; CL2355, dvIs50 [pCL45 (snb-1::Abeta 1-42::3′UTR(long) + mtl-2::GFP]; CL2006, dvIs2 [pCL12(unc-54/human Abeta peptide 1-42 minigene) + rol-6(su1006)]; dvIs2179 [*myo-3p*::GFP::3′UTR(long) + rol-6(su1006)]; AM141, rmIs133 [unc-54p::Q40::YFP]; CF1553, muIs84 [(pAD76) *sod-3p*::GFP + rol-6(su1006)]; CL2166, dvIs19 [(pAF15)*gst-4p*::GFP::NLS] III; TJ375, gpIs1 [*hsp-16.2p*::GFP]. CL4176, CL2355, and CL2122 were cultured at 15 °C, and the rest of the strains were cultured at 20 °C. All nematodes were incubated on nematode growth medium (NGM) plates seeded with *Escherichia coli* OP50 as food resources. Pregnant adult nematodes were treated with the mixture of sodium hypochlorite and NaOH to obtain eggs and then synchronized L1 stage larvae were obtained after 24 h incubation in M9 buffer.

### 4.2. Isolation of Cryptotanshinone from Salvia Castanea and Treatment

The whole plant of *Salvia castanea* was collected from Lijiang city, Yunnan Province, China, in June 2018, and the plant material was identified by Dr. Dong-Qing Fei of the School of Pharmacy, Lanzhou University. A voucher specimen (no. 201806SC) was deposited in the School of Pharmacy, Lanzhou University. 

The air-dried whole plant of *S. castanea* (7.5 kg) was powdered and extracted with 95% ethanol four times at room temperature (7 days each time). The solvent was removed under reduced pressure to afford a dark brown crude extract (1400 g). The extract was suspended in H_2_O and partitioned with EtOAc to yield an EtOAc extract (528 g). The EtOAc extract (528 g) was fractionated by silica gel (200–300 mesh) column chromatography eluted with petroleum ether-acetone (40:1–1:1) to give six fractions (Fr.1–Fr.6). Fr.3 (75 g) was applied to an MCI gel column eluted with aqueous EtOH (30–100%) to yield five subfractions (Fr.3.1–Fr.3.5). Fr.3.2 (15 g) was separated by silica gel column chromatography with CHCl_3_-acetone (60:1–1:1) and further purified on a Sephadex LH-20 column using CHCl_3_-CH_3_OH (2:3) as an eluent to afford cryptotanshinone (60 mg). 

Cryptotanshinone was dissolved in DMSO to 20 mM and stored at 4 °C as a master stock solution. The final concentration of DMSO cannot exceed 0.1% (*v*/*v*) after it is added to NGM. Vehicle control NGM plates contained equivalent volumes of DMSO. Ultimately, the CTS concentration in the NGM was L (2 µM), M (10 µM), and H (20 µM).

### 4.3. Paralysis Assay

The paralysis assay of the nematode CL4176 was completed as previously described [52]. The synchronized L1 stage larvae were counted and spread to the NGM with or without CTS. Each 30 mm plate was inoculated with 50–60 nematodes. The larvae were cultured at 15 °C to the L3 stage and then the temperature was raised to 25 °C to induce Aβ expression and accumulation. After 34 h, the number of paralyzed nematodes was counted every two hours until 80% of the paralysis was in the control. The whole body being unable to move or only head movement was recorded as paralysis. The experiments were repeated at least three times.

### 4.4. Sensitivity Assay of Exogenous Serotonin

The CL2355 strain expressed human Aβ peptide in pan-neurons and was hypersensitive to exogenous serotonin [53]. CL2122 is a transgenic control strain of CL2355 and has no Aβ expression in its neurons. Synchronized L1 larvae were treated with or without CTS at 15 °C for 72 h, then transferred to 25 °C for another 36 h, respectively. The worms were collected and washed with M9 buffer. In total, 30 worms in each group were transferred to 150 µL M9 buffer containing 5 mg/mL serotonin. After 5 min, the paralyzed worms were scored. The inability of worms to wriggle within 5 s is considered paralysis. The experiment completed three biological replications.

### 4.5. Fluorescence Staining of Aβ Deposits

Thioflavin S (ThS, Sigma, St Louis, MO, USA) staining was performed to detect Aβ deposits in *C. elegans* [54]. The synchronized L1 larvae of CL2006 were inoculated on NGM for 4 days, then transferred to fresh NGM with or without CTS for 2 days. The *C. elegans* was washed three times with M9 buffer and fixed with 4% paraformaldehyde (pH 7.4) at 4 °C for 24 h. After that, the samples were permeabilized using permeabilization buffer (5% β-mercaptoethanol, 1% Triton X-100, and 125 mM Tris-HCl, pH 7.4) at 37 °C for 24 h. The samples were washed three times with PBST and stained with ThS (0.125%, dissolution in 50% ethanol). After decolorization with 50% ethanol, PBS was added and observed under a fluorescence microscope (DS-Ri2; Nikon, Tokyo, Japan). At least 30 images were taken in each group. The anterior pharyngeal area was measured with ImageJ, and the number of deposition spots was counted. The experiment was repeated three times.

### 4.6. Western Blotting

The expression of human Aβ_1−42_ in the transgenic *C. elegans* CL4176 was detected by Western blotting [38,55,56]. Nematodes were washed with M9 buffer and stored at −80 °C for 2 h, then homogenized in lysis buffer containing protease inhibitor (Sigma, St Louis, MO, USA). After boiling for 10 min at 100 °C and sitting on ice for 30 min, the samples were centrifuged at 14,000× *g* for 10 min at 4 °C. The supernatant was transferred to new 1.5 mL centrifuge tubes. The protein solution added loading buffer (Solarbio, Beijing, China) in each group was heated at 100 °C for 5 min to denature it. After cooling, the samples were divided and stored at −20 °C. Equal totals of protein solution were loaded on Tris-Tricine gel for electrophoresis. The protein was transferred from the gel to 0.22 µm PVDF membrane, and then the membrane was blocked with 5% milk at room temperature for 2 h. The primary antibody of 6E10 (Biolegend, CA, USA) was incubated overnight at 4 °C at 1:2000 dilution. Goat anti-mouse HRP was used as a secondary antibody at 1:10,000 dilution and incubated for 1.5 h at room temperature. β-actin (Affinity Biosciences, Jiangsu, China), used as an internal reference at 1:5000 dilution, was incubated overnight at 4 °C, too. Finally, bands of protein were visualized by ECL chemiluminescence. ImageJ software was used to analyze the relative band intensities of Aβ monomers and oligomers and calculated the intensities value normalized to the intensity values of β-actin.

### 4.7. Measurement of ROS Level and SOD Activity in CL4176 Nematodes

According to the previous report [57,58], synchronized L1 stage CL4176 nematodes were inoculated on OP50/NGM plates containing DMSO/CTS and incubated at 15 °C for 72 h to L3 stage, then transferred to 25 °C for 36 h [59]. The nematodes were collected in M9 buffer, washed off of the residual OP50, homogenized by adding PBST-containing PMSF, and centrifuged. For ROS detected, a black 96-well plate was incubated with 50 μL of the sample extract and 50 μL of 200 μM DCFH-DA probe (Meilunbio, Dalian, China) for 30 min at 25 °C. The fluorescence intensity was measured using a multi-mode microplate reader (Thermo Scientific, Waltham, MA, USA) at an excitation wavelength of 504 nm and an emission wavelength of 529 nm. The experiment was repeated three times at 20 min intervals for two hours. 

For the SOD activity detected, the reagents and samples were added to the 96-well plate according to the kit’s instructions (Beyotime Biotechnology, Shanghai, China), incubated at 37 °C for 30 min, and the absorbance was measured at 560 nm using a Microplate Reader. In the two assays, the total protein concentrations were quantified using the BCA kit (Solarbio, Beijing, China) to balance the differences in the sample volumes.

### 4.8. In Vitro Antioxidant Assay

The free radical scavenging capacity measurement (DPPH method): 5 μL CTS/DMSO solution and 150 μL 50 μM DPPH methanol solution were added to 96-well plates. The final concentrations of CTS were 50 μM, 25 μM, and 5 μM. The samples were allowed to stand for 15 min at room temperature, protected from light, and the absorbance was measured at 490 nm using a microplate reader. The total free radical scavenging (%) = (A_C_ − A_S_)/A_C_ × 100% (A_C_ is the absorbance at 490 nm of the control, A_S_ is the absorbance at 490 nm of the sample).

The total antioxidant capacity test (ABTS method): The in vitro antioxidant capacity of CTS was determined based on the total antioxidant capacity assay kit (Beyotime Biotechnology, Shanghai, China). The total antioxidant capacity (%) = (A_C_ − A_S_)/A_C_ × 100% (A_C_ is the absorbance of the blank control at 734 nm; A_S_ is the absorbance of the sample at 734 nm).

### 4.9. Subcellular Localization Assay of DAF-16

The synchronized L1 larvae were seeded onto NGM with or without CTS and maintained for 72 h at 20 °C, then collected and washed three times with M9 buffer. TJ356 incubated at 37 °C for 30 min was used as the positive control. A drop of the nematode was added to the glass slide, followed by a drop of 20 mM sodium azide, covered with a coverslip, and the fluorescent images were observed under a fluorescence microscope (DS-Ri2; Nikon, Tokyo, Japan). Thirty images were obtained at random, and the percentage of them in which translocation occurred was counted. Three experimental replications were performed.

### 4.10. Quantitative Analysis of myo-3p::GFP, Q40::YFP, hsp-16.2p::GFP, sod-3p::GFP, Gst-4p::GFP

To determine whether CTS specifically reduces the levels of Aβ in muscle cells, this test was performed [60]. CL2179 was maintained and treated in the same way as the paralysis assay. At the 36th hour after raising the temperature to 25 °C, the nematodes were collected, and the OP50 was washed off with M9 buffer. The nematodes were transferred to a glass side, and a drop of sodium azide was added (20 mM), then it was covered with a coverslip. The fluorescence intensity was obtained under the fluorescence microscope (DS-Ri2; Nikon, Tokyo, Japan). 

Respectively, after synchronization, AM141, TJ375, CF1553, and CL2166 were inoculated onto NGM with or without CTS for 72 h at 20 °C. The nematodes were collected with M9 buffer and washed off OP50. After dropping on a slide with sodium azide, the number of Q40::YFP fluorescent spots in AM141 nematodes was observed and counted with a fluorescence microscope (DS-Ri2; Nikon, Tokyo, Japan), and the corresponding fluorescence intensity was measured in all other strains. Each group took at least 30 images and repeated the experiment three times independently.

### 4.11. Analysis of the Expression Levels of Related Genes

After CL4176 was synchronized, the L1 stage larvae were inoculated onto NGM with or without CTS, incubated at 15 °C for 72 h, and transferred to 25 °C for further incubation for 30 h [50]. Then, the nematodes were collected with M9 buffer and washed to remove the residual OP50. The total RNA of each group was extracted using the TRIeasy^TM^ Total RNA Extraction Reagent (Yeasen Biotechnology, Shanghai, China). The concentration of total RNA was detected by NanoDrop One (Thermo Scientific, Waltham, MA, USA). The cDNA was reverse transcribed according to the instructions of Hifair^®^ III 1st Strand cDNA Synthesis SuperMix for qPCR (Yeasen Biotechnology, Shanghai, China) and stored at −20 °C. For real-time PCR amplification using Hieff^®^ qPCR SYBR^®^ Green Master Mix (Yeasen Biotechnology, Shanghai, China), the reactions were performed on QuantStudio™ 3 (ABI, Waltham, MA, USA). Three biological replicates were used for each sample. The relative expression of each gene was analyzed by the 2^-ΔΔCt^ method, using the actin gene as an internal reference. The forward and reverse primers used in the experiment are shown in Table 1.

### 4.12. Statistical Analysis

SPSS 23.0 software was used to perform the statistical analysis of the data, and GraphPad 9.0 software was used for graphing. The data form was average ± standard deviation (Mean ± SD). One-way ANOVA analysis was used to evaluate the significant difference among groups. The survival analysis was used to analyze the data of the paralysis assay. Kaplan–Meier and log-rank tests were used to analyze the differences between the groups. *p* < 0.05 was considered to have statistical significance.

## 5. Conclusions

The combined effect of antioxidants and the reduction in toxic Aβ aggregates is necessary to mitigate Aβ-induced toxicity. Compounds that inhibit Aβ aggregation and exhibit significant antioxidant activity can be considered effective therapeutic agents for AD [61]. In summary, the present study fully demonstrates that CTS can reduce oxidative stress and lower abnormal aggregated protein levels in nematodes by exerting effects on multiple pathways, such as the upregulation of HSPs levels, the enhancement of SOD activity, and the inhibition of *ace-2*. It has the potential to be developed as a therapeutic agent for AD, but the specific mechanism needs to be further investigated subsequently.

## Figures and Tables

**Table 1:** Primers for target genes and internal reference genes.

Genes	Primers	
*Aβ*	F:5′-CCGACATGACTCAGGATATGAAGT-3′	R:5′-CACCATGAGTCCAATGATTGCA-3′
*ace-1*	F:5′-AGTGGGCTCCTGTTCGAGAA-3′	R:5′-CCAATAGAAAATCACCATCGACAA-3′
*ace-2*	F:5′-CAATAATCAACTCATGGGCATCA-3′	R:5′-TTTTCGCGAGACGAAACGA-3′
*bec-1*	F:5′-ACGAGCTTCATTCGCTGGAA-3′	R:5′-TTCGTGATGTTGTACGCCGA-3′
*daf-16*	F:5′-ACCGTTGGTCAAATGCTTGC-3′	R:5′-TGGCTTCTTACGACAACGCT-3′
*hsf-1*	F:5′-ATGCAGCCAGGATTGTCGAA-3′	R:5′-GCACGTTTTGAGTTGGGTCC-3′
*skn-1*	F:5′-GAGAGAAGGGCACACGACAA-3′	R:5′-TCGAGCATTCTCTTCGGCAG-3′
*gst-4*	F:5′-GCTGAAGCCAACGACTCCAT-3′	R:5′-GACCGAATTGTTCTCCATCGA-3′
*sod-1*	F:5′-CGTAGGCGATCTAGGAAATGTG-3′	R:5′-AACAACCATAGATCGGCCAACG-3′
*sod-2*	F:5′-AGCTTTCGGCATCAACTGTC-3′	R:5′-AAGTCCAGTTGTTGCCTCAAGT-3′
*sod-3*	F:5′-TTCAAAGGAGCTGATGGACACT-3′	R:5′-AAGTGGGACCATTCCTTCCAA-3′
*sod-4*	F:5′-GTTGTCTAAGTGCTGGTGG-3′	R:5′-TTCCACATGCAAGTCGGCT-3′
*sod-5*	F:5′-GCAAAATGAATCATGGAGGAAG-3′	R:5′-AAGATCATCTCGATCGACGTGG-3′
*tnfaip1*	F:5′-CCAGAAGAATCCCCATACGA-3′	R:5′-TCCTCCTCCAACTTTTCCAAA-3′
*tnfaip*	F:5′-TCCCCATACGAAACAACACA-3′	R:5′-CTCCTCCCAGCTTTTCCACAA-3′
*actin*	F:5′-CCACGTCATCAAGGAGTCAT-3′	R:5′-GGAAGCGTAGAGGGAGAGGA-3′

## Data Availability

The data used to support the findings of this study are available from the corresponding author upon reasonable request.
